# Editorial: Autoantibodies for diagnostics, prognostics, and surveillance in autoimmune disease

**DOI:** 10.3389/fmed.2024.1491035

**Published:** 2024-09-20

**Authors:** Helena Enocsson, Ingrid E. Lundberg, Martin Herrmann, Ioannis Parodis, Christopher Sjöwall

**Affiliations:** ^1^Division of Inflammation and Infection/Rheumatology, Department of Biomedical and Clinical Sciences, Linköping University, Linköping, Sweden; ^2^Division of Rheumatology, Department of Medicine Solna, Karolinska Institutet and Karolinska University Hospital, Stockholm, Sweden; ^3^Department of Internal Medicine 3-Rheumatology and Immunology, Friedrich-Alexander-Universität Erlangen-Nürnberg (FAU) and Universitätsklinikum Erlangen, Erlangen, Germany; ^4^Deutsches Zentrum für Immuntherapie (DZI), Friedrich-Alexander-Universität Erlangen-Nürnberg (FAU) and Universitätsklinikum Erlangen, Erlangen, Germany; ^5^Department of Rheumatology, Faculty of Medicine and Health, Örebro University, Örebro, Sweden

**Keywords:** autoantibodies, autoimmunity, rheumatoid arthritis, autoimmune liver disease (ALD), myositis, antiphospholipid syndrome, antinuclear antibodies (ANA), type I IFN

Breach of tolerance to self-antigens and serological autoimmunity, reportedly occurs long before onset of clinical autoimmune disease (1). Still, circulating disease-specific autoantibodies remain a hallmark of B-cell driven autoimmune disease although they may also be detected in healthy individuals and transiently in patients with infections. Over the years associations between specific autoantibodies and clinical disease have improved our understanding of mechanisms in autoimmunity as well as clinical diagnostics (2, 3).

Lack of immune tolerance and increased production of antibodies directed against self-antigens may induce or precipitate autoimmune disease as clearly demonstrated for the TSH receptor antibodies in Graves' disease. Beyond that, autoantibodies may represent an epiphenomenon in autoimmune disease without distinct pathophysiologic relevance; this is exemplified by smooth muscle antibodies (SMA) in autoimmune hepatitis (AIH). However, most autoimmune diseases, e.g., systemic lupus erythematosus (SLE), idiopathic inflammatory myopathies (IIM), and Sjögren's disease (SjD) are characterized by mixtures of autoantibodies with varying degrees of pathogenic potential. They can help physicians to make a correct diagnosis or to predict the severity of the disease or the response to treatments. In addition, “new” autoantibodies with clinical relevance are constantly discovered and new areas of application for “old” autoantibodies are emerging (4).

The Research Topic “*Autoantibodies for Diagnostics, Prognostics, and Surveillance in Autoimmune Disease*” collected original works, review articles and case reports offering new insights on clinical applications of new and established autoantibodies in autoimmune diseases. The clinical use of autoantibodies has the potential to facilitate early diagnosis, informed selection of patients for targeted and personalized therapies which in turn can be expected to improve long-term outcomes for patients with autoimmune diseases. Furthermore, the discovery of (new) autoantibodies and their autoantigens may give clues to disease mechanisms and reveal new areas of research. We hereby summarize the final content of the Research Topic which covers a variety of autoimmune diseases and autoantibodies.

In a single center study by Marklund et al. the presence of myositis-specific autoantibodies (MSA) and myositis associated autoantibodies (MAA) were examined in relation to lung involvement in patients with myositis. Patients presenting with MAA, particularly anti-SSA/Ro52 antibodies were found to be at higher risk of developing interstitial lung disease (ILD). Interestingly, it was also noticed that patients presenting only with antibodies against Mi-2α, Mi-2β, NXP2, HMGCR, and TIF1γ, or no MSA/MAA, were all spared from ILD.

Zhang et al. contributed a case report describing a patient with anti-MDA5 positive amyopathic dermatomyositis, also positive for anti-SSA/Ro52. This patient, who suffered from rapidly progressive ILD was successfully treated with methylprednisolone pulses combined with cyclosporine A and hydroxychloroquine.

In a review, Antiochos and Casciola-Rosen thoughtfully described the autoantigens associated with the interferon (IFN) system. They included the type I IFN induced autoantigen SSA/Ro52, as well as MDA-5, a cytoplasmic dsRNA sensor that promote type I IFN production. The authors also describe autoantibodies against IFN types I, II and III themselves, and linked them to autoimmune diseases and immunodeficiencies.

A cross-sectional study by Sciascia et al. investigated autoantibodies recognizing high density lipoprotein (HDL) in antiphospholipid syndrome (APS). Patients with APS showed higher levels of anti-HDL when compared to healthy controls; the highest levels were found among patients with arterial, as compared to venous thrombosis, even when adjusting for total IgG. The results suggest that whether anti-HDL autoantibodies could serve a biomarker for thrombotic events in APS deserve further studies.

Andraos et al. assessed a large collection of sera from healthy blood donors (*n* = 825) for ANA using both HEp-2 cells and ALBIA. A considerable proportion of the sera contained autoantibodies, though without any clear association to self-reported symptoms. Importantly, the combination of ANA fine-specificities, relevant symptoms and high IFN-α levels identified a small proportion of blood donors with autoimmune disease (two cases with SjD).

In a case-control study by Liu et al., the presence, titer and pattern of antinuclear antibodies (ANA) among patients with newly diagnosed rheumatoid arthritis (RA) were investigated. ANA positivity, especially at high titers and homogenous staining pattern on Hep-2 cells, was associated with a higher probability of RA when compared with healthy individuals and non-RA subjects with arthritis.

Another study, by Martinsson et al., examined the relation between a periodontitis pathogen (*Aggregatibacter actinomycetemcomitans*; A.a.) and the development of RA in at-risk RA patients from two cohorts. Leukotoxin A (LtxA) is expressed by A.a. and has the indirect ability of citrullination, thereby providing a possible route for an increased load of citrullinated antigens. These may induce anti-citrullinated protein antibodies (ACPA), a hallmark of RA. An association between anti-LtxA antibodies with disease progression to RA was found in one of the two cohorts, but not in the other. The authors suggest that there might be population-dependent differences in the oral microbiome and the risk to develop RA, which should be further explored in future studies.

A review article by Wang et al. covered the area of Chinese herbal medicine and the beneficial effects of various herbs and herbal compounds in the treatment of RA. A structured overview of these compounds, their biological targets and potential mechanism of action were given. The focus was on anti-inflammatory and antioxidative effects. This review provides inspiration for further research into herbal remedies that, in the future, may expand treatment options for patients with RA.

Van den Beukel et al. a investigated autoantibodies against six post-translationally modified (PTM) proteins in patients with autoimmune liver disease. Anti-PTM were more frequent among patients compared to healthy controls and presence of multiple anti-PTM was more frequently found in the subgroup of patients with autoimmune hepatitis (AIH). These patients also displayed a higher rate of complete biochemical response to treatment, defined as normalized IgG and aminotransferases. In a commentary to this contribution, Taubert et al. described polyreactivity of IgG to multiple autoantigens highlighting the risk of false positive results when measuring autoantibodies in sera from patients with AIH (5). The polyreactivity includes bovine serum albumin a canonical ELISA blocking reagent (5, 6). Van den Beukel et al. responded to this commentary by assuring that appropriate control antigens have been used in their ELISA assays. It was exemplified by a deepened description of their detection of autoantibodies to carbamylated proteins.

In summary, our Research Topic provides valuable insights into autoantibodies and autoimmune diseases, with emphasis on novel applications of known autoantibodies. We are convinced that these contributions will aid in improving clinical diagnostics, prognostication and patient management as well as inspire further research.
